# Clinicopathological Determinants of an Elevated Systemic Inflammatory Response Following Elective Potentially Curative Resection for Colorectal Cancer

**DOI:** 10.1245/s10434-017-5987-z

**Published:** 2017-07-10

**Authors:** David G. Watt, Michelle L. Ramanathan, Stephen T. McSorley, Killian Walley, James H. Park, Paul G. Horgan, Donald C. McMillan

**Affiliations:** 1 Academic Unit of Surgery, R2.06, School of Medicine—University of Glasgow, Glasgow, UK; 2 Department of Surgery, Glasgow Royal Infirmary, Glasgow, UK

## Abstract

**Introduction:**

The postoperative systemic inflammatory response (SIR) is related to both long- and short-term outcomes following surgery for colorectal cancer. However, it is not clear which clinicopathological factors are associated with the magnitude of the postoperative SIR. The present study was designed to determine the clinicopathological determinants of the postoperative systemic inflammatory response following colorectal cancer resection.

**Methods:**

Patients with a histologically proven diagnosis of colorectal cancer who underwent elective, potentially curative resection during a period from 1999 to 2013 were included in the study (*n* = 752). Clinicopathological data and the postoperative SIR, as evidenced by postoperative Glasgow Prognostic Score (poGPS), were recorded in a prospectively maintained database.

**Results:**

The majority of patients were aged 65 years or older, male, were overweight or obese, and had an open resection. After adjustment for year of operation, a high day 3 poGPS was independently associated with American Society of Anesthesiologists (ASA) grade (hazard ratio [HR] 1.96; confidence interval [CI] 1.25–3.09; *p* = 0.003), body mass index (BMI) (HR 1.60; CI 1.07–2.38; *p* = 0.001), mGPS (HR 2.03; CI 1.35–3.03; *p* = 0.001), and tumour site (HR 2.99; CI 1.56–5.71; *p* < 0.001). After adjustment for year of operation, a high day 4 poGPS was independently associated with ASA grade (HR 1.65; CI 1.06–2.57; *p* = 0.028), mGPS (HR 1.81; CI 1.22–2.68; *p* = 0.003), NLR (HR 0.50; CI 0.26–0.95; *p* = 0.034), and tumour site (HR 2.90; CI 1.49–5.65; *p* = 0.002).

**Conclusions:**

ASA grade, BMI, mGPS, and tumour site were consistently associated with the magnitude of the postoperative systemic inflammatory response, evidenced by a high poGPS on days 3 and 4, in patients undergoing elective potentially curative resection for colorectal cancer.

Colorectal cancer is the second most common cause of cancer death in the United Kingdom, accounting for 16,000 deaths annually.^1^ It has been recognised that the preoperative systemic inflammatory response is related to long-term outcome in patients following potentially curative surgery for colorectal cancer.^2^–^4^

It also has been reported that the postoperative systemic inflammatory response can be a useful early predictor of the development of postoperative infective complications and anastomotic leak following colorectal cancer resection, regardless of surgical approach.^5^–^8^ Indeed, C-reactive protein (CRP) concentrations >150 mg/L on postoperative days 3–5 have been consistently reported to be associated with postoperative complications.^9^ With this in mind, a postoperative systemic inflammation score, based on the combination of CRP and albumin was developed and termed the postoperative Glasgow Prognostic Score (poGPS). This score recently has been reported to predict the development and severity of postoperative infective complications as well as long-term survival.^10^

With the expansion in the use of enhanced recovery after surgery (ERAS) programmes to improve short-term outcomes following surgery and promote timely discharge, ERAS programmes have been proposed to reduce the surgical stress response. However, in a recent systematic review, it was concluded that of the components of an ERAS programme, only the use of laparoscopic surgery was consistently associated with a lower postoperative systemic inflammatory response.^11^ Therefore, clinicopathological factors that influence the postoperative systemic inflammatory response, as evidenced by the poGPS, are of considerable interest, because they may be modifiable and therefore potentially could be considered as future therapeutic targets. The purpose of the present study was to examine the clinicopathological determinants of the postoperative systemic inflammatory response, as evidenced by the poGPS on postoperative days 3 and 4, in patients following resection of colorectal cancer.

## Methods

Patients with a histologically proven diagnosis of colorectal cancer who, based on preoperative investigations and operative findings, were considered to have undergone potentially curative resection at a single centre during a period from 1999 to 2013 were initially included in the study (*n* = 834). All procedures were performed at Glasgow Royal Infirmary, a University teaching hospital where procedures were performed by consultant surgeons with a subspecialist interest in colorectal surgery or by trainees who were supervised by these consultants. Patient characteristics, including perioperative C-reactive protein concentrations, were recorded routinely in a prospective departmental audit database. All patient data were anonymised.

All tumours were staged according to the conventional tumour, node, metastasis (TNM, 5th edition) classification. Patients with metastatic disease were excluded from analysis. Daily blood samples were obtained, as per hospital routine, during the perioperative period and were standard care for all patients. Before surgery, all patients received thromboprophylaxis and antibiotic prophylaxis as per hospital policy at the time. Lesions from the caecum to the sigmoid colon were classified as colon cancers, lesions of the rectosigmoid junction and rectum were classified as rectal cancers.

The preoperative systemic inflammatory response was assessed using the modified Glasgow Prognostic Score (mGPS), an extensively validated and independently prognostic systemic inflammation based score. Briefly, mGPS patients with a normal C-reactive protein (<10 mg/L) were allocated a score of 0, those with an elevated C-reactive protein (>10 mg/L) allocated a score 1, and those with an elevated C-reactive protein (>10 mg/L) and hypoalbuminaemia (<35 g/L) were allocated a score of 2.

The poGPS was calculated as previously described by Watt et al.^19^ and is as follows: postoperative CRP concentration <150 mg/L, regardless of albumin concentration, scored 0; CRP concentration >150 mg/L and albumin >25 g/L scored 1; and CRP >150 mg/L and albumin <25 g/L scored 2. Emergency presentation was determined if the patient presented via an unplanned hospital admission and underwent surgery during the same admission.

Patient comorbidity was classified using the American Society of Anesthesiologists (ASA) grading system: 1 represents a normal healthy patient, 2 is a patient with mild systemic disease, 3 is a patient with severe systemic disease, and 4 is a patient with severe systemic disease that is a constant threat to life. This assessment was performed by an anaesthetist preoperatively. Body mass index (BMI) was categorised as normal weight (<25), overweight (≥25–30), and obese (>30). BMI was obtained from the patients’ electronic preoperative assessment record and ASA grade from the Opera Theatre Management system (OPERA v4.0, CHCA, Canada). The study was approved by the West of Scotland Research Ethics Committee, Glasgow.

### Statistical Analysis

The relationship between clinicopathological variables and days 3 and 4 poGPS were examined using the χ^2^ test for categorical variables. Binary logistic regression was used to examine the relationship between clinicopathological factors and the presence of a postoperative systemic inflammatory response, indicated by a poGPS score of >1 on both days 3 and 4 and calculate an OR and 95% CI. Clinicopathological factors that on univariate analysis had a *p* value <0.10 were taken into a multivariate model using a backward conditional model to identify independently significant factors. A *p* value of <0.05 was considered significant. Statistical analysis was performed using SPSS version 22.0 for Windows (IBM Corporation, Armonk, NY).

## Results

Baseline characteristics of the 752 patients who underwent surgery for colorectal cancer are shown in Table 1. The majority of patients were aged 65 years or older (64%), male (54%), were overweight or obese (58%), had colonic tumours (62%), were not systemically inflamed (68% mGPS 0; 52% NLR ≤3), and had node-negative disease (63%). Most patients underwent open resection (85%). Overall, 23% of patients developed an infective complication.

**Table 1:** Clinicopathological characteristics of patients undergoing elective potentially curative, elective resection for colorectal cancer (*n* = 752)

Characteristic	No. of patients (%)
Age (<65/65–74/≥ 75)	245 (33)/272 (33)/235 (31)
Sex (female/male)	344 (46)/408 (54)
Year of operation (1999–2006/2007–2013)	311 (41)/408 (54)
ASA (1/2/3/4)^a^	58 (11)/214 (42)/219 (42)/24 (5)
BMI (<25/> 25–30/>30)^b^	186 (34)/223 (41)/131 (24)
TNM stage (0/I/II/III)	12 (2)/141 (19)/321 (43)/278 (37)
T stage (I/II/III/IV)	62 (8)/106 (14)/411 (55)/161 (21)
N stage (0/I/II)	474 (63)/206 (27)/72 (10)
Tumour site (colon/rectum)	467 (62)/285 (38)
Laparoscopic (no/yes)	640 (85)/112 (15)
Preop mGPS	498 (68)/134 (18)/100 (14)
Preop NLR (≤3/>3)	391 (52)/358 (48)
Day 3 poGPS (0/1/2)	361 (53)/186 (27)/135 (20)
Day 4 poGPS (0/1/2)	439 (72)/87 (14)/86 (14)
Infective complications (no/yes)	580 (77)/169 (23)

*ASA* American Society of Anesthesiology Grading System; *BMI* body mass index; *mGPS* modified Glasgow Prognostic score; *TNM* Tumour Node Metastases; *CRP* C-reactive protein

^a^ *n* = 516

^b^ *n* = 540

The relationships between days 3 and 4 poGPS and clinicopathological characteristics are shown in Table 2. The day 3 poGPS was significantly associated with male sex (*p* < 0.05), later year of operation (*p* < 0.001), ASA grade (*p* < 0.01), BMI (*p* < 0.001), mGPS (*p* < 0.001), preop NLR (*p* < 0.001), rectal cancer (*p* < 0.01), laparoscopic surgery (*p* < 0.01), and T stage (*p* < 0.01). The day 4 poGPS was significantly associated with later year of operation (*p* < 0.001), BMI (*p* < 0.05), mGPS (*p* < 0.01), NLR (*p* < 0.005), and laparoscopic surgery (*p* < 0.005). Moreover, when year of operation was further divided into three groups (1999–2003; 2004–2008; 2009–2013), the poGPS of both day 3 and day 4 remained significantly associated with year of operation (both *p* < 0.001). The day 3 poGPS was significantly associated with an increase in infective complications from 12 to 31 to 45%, and the day 4 poGPS was significantly associated with an increase in infective complication rates from 16 to 39 to 58%.

**Table 2:** The relationship between clinicopathological characteristics and postoperative days 3 and 4 poGPS in patients undergoing elective, potentially curative surgery for colorectal cancer (*n* = 752)

Characteristic		n	Day 3 poGPS (0/1/2)	*p* value	Day 4 poGPS (0/1/2)	*p* value
Age (year)				0.247		0.662
	<65	245	117 (53)/62 (28)/41 (19)		135 (70)/28 (14)/31 (16)	
	65–74	272	120 (49)/76 (31)/47 (19)		155 (70)/35 (16)/30 (14)	
	≥75	235	124 (57)/48 (22)/47 (21)		149 (75)/24 (12)/25 (13)	
Sex				0.012		0.109
	Female	344	181 (57)/69 (22)/66 (21)		200 (75)/29 (11)/38 (14)	
	Male	408	180 (49)/117 (32)/69 (19)		239 (69)/58 (17)/48 (14)	
Year of operation				<0.001		<0.001
	1999–2006	311	160 (58)/87 (32)/28 (10)		196 (82)/28 (12)/14 (6)	
	2007–2013	441	201 (49)/99 (24)/107 (26)		243 (65)/59 (16)/72 (19)	
Year of operation				<0.001		<0.001
	1999–2003	172	89 (61)/49 (33)/9 (6)		102 (84)/15 (12)/5 (4)	
	2004–2008	228	113 (52)/60 (28)/43 (20)		148 (76)/22 (11)/24 (12)	
	2009–2013	352	159 (50)/77 (24)/83 (26)		189 (64)/50 (17)/57 (19)	
ASA				0.008		0.446
	1	58	37 (72)/8 (16)/6 (12)		30 (79)/5 (13)/3 (8)	
	2	214	109 (56)/64 (33)/22 (11)		132 (75)/28 (16)/15 (9)	
	3	219	107 (51)/57 (27)/44 (21)		137 (71)/30 (16)/25 (13)	
	4	24	7 (33)/7 (33)/7 (33)		15 (68)/2 (9)/5 (23)	
BMI				<0.001		0.003
	<25	186	103 (60)/33 (19)/35 (21)		97 (70)/15 (11)/26 (19)	
	>25–30	223	107 (53)/70 (35)/23 (12)		138 (73)/35 (19)/15 (8)	
	>30	131	45 (40)/39 (34)/29 (26)		62 (59)/25 (24)/18 (17)	
mGPS				<0.001		0.007
	0	498	260 (56)/128 (28)/75 (16)		302 (74)/54 (13)/50 (12)	
	1	134	50 (41)/45 (37)/26 (22)		71 (68)/21 (20)/13 (12)	
	2	100	43 (48)/13 (14)/34 (38)		55 (61)/12 (13)/23 (26)	
NLR				<0.001		0.002
	≤3	391	177 (49)/122 (33)/66 (18)		216 (66)/60 (18)/51 (16)	
	>3	358	184 (59)/62 (20)/68 (21)		222 (78)/27 (10)/34 (12)	
Tumour site				0.009		0.076
	Colon	467	225 (54)/126 (30)/69 (16)		264 (72)/58 (16)/43 (12)	
	Rectum	285	136 (52)/60 (23)/66 (25)		175 (71)/29 (12)/43 (17)	
Laparoscopic				0.006		0.002
	No	640	299 (51)/158 (27)/127 (22)		390 (73)/66 (12)/79 (15)	
	Yes	112	62 (63)/28 (29)/8 (8)		49 (64)/21 (27)/7 (9)	
T stage				0.007		0.578
	I	62	23 (42)/26 (47)/6 (11)		31 (63)/11 (22)/7 (14)	
	II	106	61 (62)/23 (24)/14 (14)		62 (71)/16 (16)/11 (13)	
	III	411	186 (50)/102 (27)/85 (23)		241 (71)/48 (14)/49 (15)	
	IV	161	84 (58)/34 (23)/27 (19)		95 (75)/14 (11)/18 (14)	
N stage				0.346		0.065
	0	474	229 (53)/109 (25)/94 (22)		275 (72)/49 (13)/59 (15)	
	I	206	100 (54)/58 (31)/29 (15)		129 (75)/27 (16)/15 (9)	
	II	72	32 (51)/19 (30)/12 (19)		35 (60)/11 (19)/12 (21)	
Infective complications				<0.001		<0.001
	No	580	316 (61)/129 (25)/74 (14)		364 (80)/53 (12)/36 (8)	
	Yes	169	42 (26)/57 (36)/61 (38)		72 (46)/34 (22)/50 (32)	

*ASA* American Society of Anesthesiology Grading System; *BMI* body mass index; *mGPS* modified Glasgow Prognostic score

Binary logistic regression of the factors significantly associated with low poGPS (poGPS 0) versus a high poGPS (poGPS 2) on day 3 and day 4 are shown in Tables 3 and 4 respectively. A high day 3 poGPS was independently associated with year of operation (hazard ratio [HR] 4.63; confidence interval [CI] 2.28–9.42; *p* < 0.001), ASA (HR 2.02; CI 1.25–3.26; *p* = 0.004), mGPS (HR 1.97; CI 1.29–3.01; *p* = 0.002), tumour site (HR 2.62; CI 1.32–5.17; *p* = 0.006), and laparoscopic surgery (HR 0.20; CI 0.05–0.73; *p* = 0.015). When year of operation was removed from the binary logistic regression analysis, only ASA grade (HR 1.96; CI 1.25–3.09; *p* = 0.003), BMI (HR 1.60; CI 1.07–2.38; *p* = 0.001), mGPS (HR 2.03; CI 1.35–3.03; *p* = 0.001), and tumour site (HR 2.99; CI 1.56–5.71; *p* < 0.001) were independently associated with a high day 3 poGPS.

**Table 3:** Binary logistic regression of clinicopathological factors associated with low poGPS (poGPS 0) versus a high poGPS (poGPS 2) on day 3 in patients undergoing elective, potentially curative resection for colorectal cancer (*n* = 752)

Characteristic	Univariate analysis		Multivariate analysis	
	HR (95% CI)	*p* value	HR (95% CI)	*p* value
Age (<65/65–74/≥75 year)	1.04 (0.81–1.32)	0.760	–	–
Sex (female/male)	1.05 (0.71–1.56)	0.804	–	–
Year of operation (99–06/07–13)	3.04 (1.91–4.84)	<0.001	4.63 (2.28–9.42)	<0.001
ASA (1/2/3/4)	1.87 (1.31–2.69)	0.001	2.02 (1.25–3.26)	0.004
BMI (<25/25–30/>30)	1.36 (1.02–1.80)	0.033	1.44 (0.94–2.20)	0.090
mGPS (0/1/2)	1.67 (1.30–2.15)	<0.001	1.97 (1.29–3.01)	0.002
NLR (≤3/>3)	0.99 (0.67–1.47)	0.965	–	–
Tumour site (colon/rectum)	1.58 (1.06–2.36)	0.024	2.62 (1.32–5.17)	0.006
Laparoscopic (no/yes)	0.30 (0.14–0.65)	0.002	0.20 (0.05–0.73)	0.015
T stage (I/II/III/IV)	1.09 (0.86–1.36)	0.482	–	–
N stage (0/I/II)	0.86 (0.63–1.18)	0.348	–	–

*ASA* American Society of Anesthesiology Grading System; *BMI* body mass index; *mGPS* modified Glasgow Prognostic score; *HR* hazard ratio; *CI* confidence interval

**Table 4:** Binary logistic regression of clinicopathological factors associated with low poGPS (poGPS 0) versus a high poGPS (poGPS 2) on day 4 in patients undergoing elective, potentially curative resection for colorectal cancer (*n* = 752)

Characteristic	Univariate analysis		Multivariate analysis	
	HR (95% CI)	*p* value	HR (95% CI)	*p* value
Age (<65/65–74/≥75 year)	0.85 (0.64–1.14)	0.285	–	–
Sex (female/male)	1.06 (0.66–1.68)	0.815	–	–
Year of operation (99–06/07–13)	4.15 (2.27–7.58)	<0.001	5.32 (2.49–11.34)	<0.001
ASA (1/2/3/4)	1.55 (1.01–2.38)	0.045	1.61 (1.02–2.55)	0.043
BMI (<25/25–30/>30)	1.03 (0.75–1.43)	0.838	–	–
mGPS (0/1/2)	1.54 (1.16–2.04)	0.003	1.98 (1.32–2.96)	0.001
NLR (≤3/>3)	0.65 (0.40–1.04)	0.073	0.47 (0.24–0.93)	0.031
Tumour site (colon/rectum)	1.51 (0.95–2.40)	0.082	2.97 (1.49–5.91)	0.002
Laparoscopic (no/yes)	0.71 (0.31–1.61)	0.408	–	–
T Stage (I/II/III/IV)	1.02 (0.79–1.32)	0.865	–	–
N Stage (0/I/II)	1.00 (0.70–1.43)	0.998	–	–

A high day 4 poGPS was independently associated with year of operation (HR 5.32; CI 2.49–11.34; *p* < 0.001), ASA (HR 1.61; CI 1.02–2.55; *p* = 0.043), mGPS (HR 1.98; CI 1.32–2.96; *p* = 0.001), NLR (HR 0.47; CI 0.24–0.93; *p* = 0.031), and tumour site (HR 2.97; CI 1.49–5.91; *p* = 0.002). When year of operation was removed from the binary logistic regression analysis, only ASA grade (HR 1.65; CI 1.06–2.57; *p* = 0.028), mGPS (HR 1.81; CI 1.22–2.68; *p* = 0.003), NLR (HR 0.50; CI 0.26–0.95; *p* = 0.034), and tumour site (HR 2.90; CI 1.49–5.65; *p* = 0.002) were independently associated with a high day 4 poGPS.

## Discussion

The results of the present study showed that, in elective surgery, year of operation, ASA grade, the preoperative modified Glasgow Prognostic Score (mGPS), and tumour site were independently associated with the postoperative systemic inflammatory response, as evidenced by the poGPS on both postoperative days 3 and 4. Therefore, in a large cohort, the present results establish the main clinicopathological factors determining an elevated systemic inflammatory response following elective potentially curative resection for colorectal cancer.

In the present study, it was of interest that the more recent time period (2007–2013) was significantly associated with a greater magnitude of the postoperative systemic inflammatory response, as evidenced by the poGPS. This was unexpected, because it might have been anticipated that with more recent surgical and anaesthetic techniques fewer patients would exceed poGPS thresholds. Indeed, patients in the more recent time period had higher BMI (*p* < 0.001) and mGPS (*p* < 0.001) values. This would suggest that elective surgery for colorectal cancer is increasingly being performed in obese and systemically inflamed patients. However, these factors were adjusted for in the present analysis, and it may be that other factors, related or unrelated, account for the greater magnitude of the postoperative systemic inflammatory response in the more recent time period.

The present results may have implications for future clinical care, particularly with respect to minimising the magnitude of the postoperative systemic inflammatory response. For example, ASA and BMI are problematic therapeutic targets in that it would be difficult to treat these directly in the relatively short time between diagnosis and elective surgery. However, the postoperative systemic inflammatory response could be targeted. For example, patients who have a high BMI or ASA may selectively receive laparoscopic surgery and perioperative care that minimises the postoperative systemic inflammatory response. Included in such care would be the use of anti-inflammatory agents such as corticosteroids.^12^,^13^ However, such targeted perioperative care is in its infancy, and the optimal timings, the agents, and the doses to minimise the magnitude of the postoperative systemic inflammatory response that will result in improved postoperative outcomes remains to be determined.^14^

In the present study, an elevated BMI was associated with an increased poGPS. To our knowledge, this relationship has not been previously reported. However, a direct relationship between BMI and C-reactive protein concentrations has been widely reported and both are established risk factors for the development of colorectal cancer.^15^ The mechanism underlying the relationship between BMI and the magnitude of the postoperative systemic inflammatory response is not clear. However, it may be that surgical injury to an increased amount of subcutaneous fat leads to a more profound systemic inflammatory response. This relationship is worthy of further study.

The laparoscopic surgical approach, compared with the open approach, has been repeatedly shown to attenuate the systemic inflammatory response.^16^–^19^ In the present study, laparoscopic surgery was independently associated with the postoperative SIR on day 3 but not day 4. The basis of this differential response to laparoscopic surgery is not clear. However, it may relate to the transient effect of this surgical approach or the interaction between laparoscopic surgery, ASA grade, and BMI. For example, patients who undergo a laparoscopic resection for colon cancer may have less comorbidity and be less obese. Indeed, it would appear that the benefit of laparoscopic surgery is more pronounced in the immediate postoperative period. The significance of this is not clear but perhaps highlights the important role the systemic inflammatory response plays early in the postoperative period. Given the increasing use of the laparoscopic approach (approximately 45% of colorectal cancer resections) these relationships warrant further study.^20^

The present study has some potential limitations that should be considered. Many preoperative factors associated with a greater postoperative systemic inflammatory response, such as obesity, diabetes, and other comorbidities, are interrelated and therefore potential confounding is an important issue in the analysis of the present study. Nevertheless, an objective measurement of the postoperative systemic inflammatory response, such as the poGPS, will facilitate in future studies the dissection of factors that have independent influence.

In conclusion, the mGPS, ASA grade, BMI, and tumour site were independently associated with a greater magnitude of the postoperative systemic inflammatory response in patients undergoing elective surgery for colorectal cancer.
